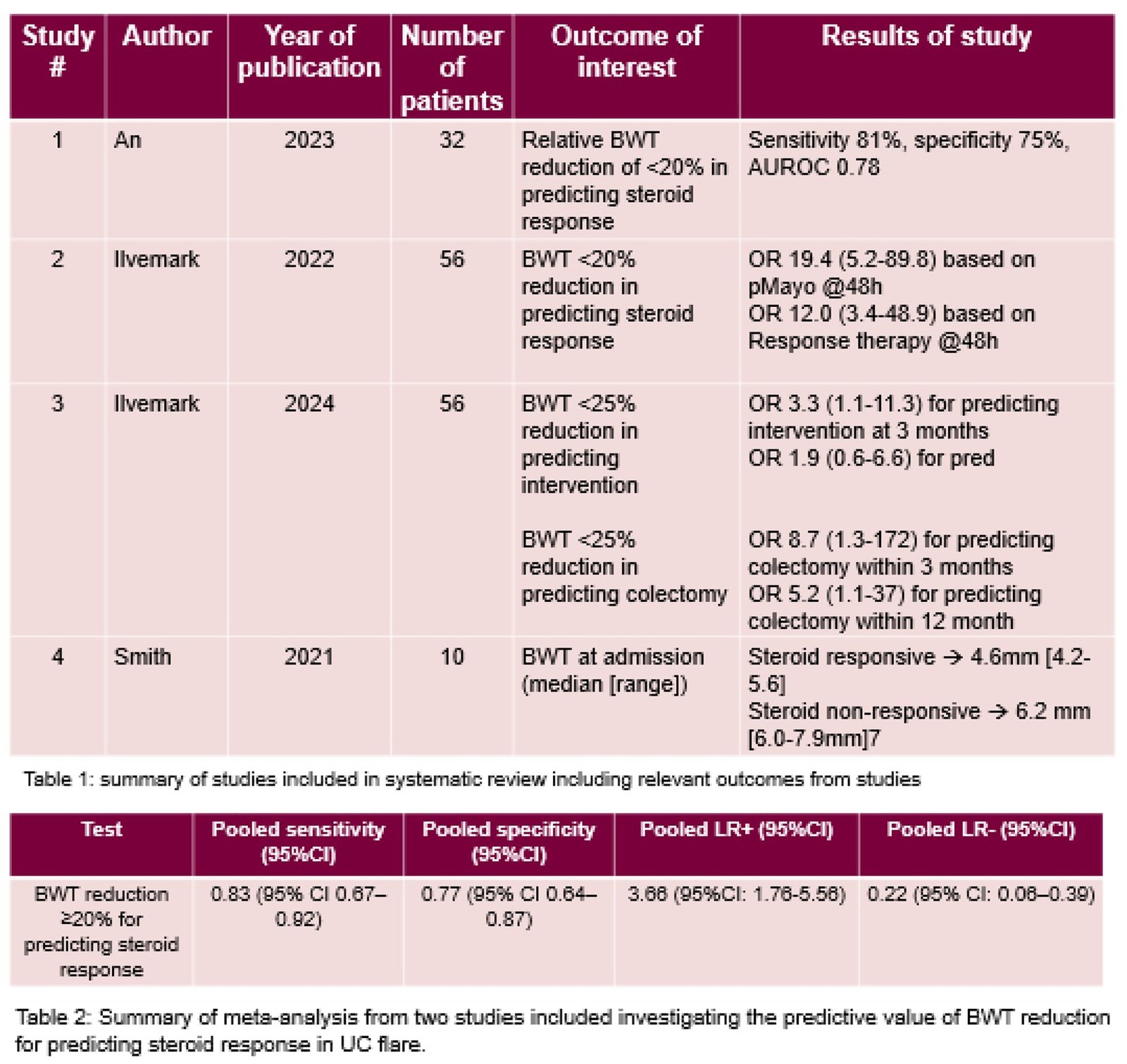# Poster Session II – Poster of Distinction II - A209 PREDICTIVE VALUE OF INTESTINAL ULTRASOUND FOR TREATMENT RESPONSE AND LONG-TERM OUTCOMES IN SEVERE ULCERATIVE COLITIS: A SYSTEMATIC REVIEW

**DOI:** 10.1093/jcag/gwaf042.209

**Published:** 2026-02-13

**Authors:** M Mohammed, S Samnani, D Borovsky, M Yaghoobi, N Calo, S Albashir

**Affiliations:** McMaster University Faculty of Health Sciences, Hamilton, ON, Canada; McMaster University Faculty of Health Sciences, Hamilton, ON, Canada; McMaster University Faculty of Health Sciences, Hamilton, ON, Canada; McMaster University Faculty of Health Sciences, Hamilton, ON, Canada; Medicine, Div of Gastroenterology, St Michael’s Hospital, Toronto, ON, Canada; McMaster University Faculty of Health Sciences, Hamilton, ON, Canada

## Abstract

**Background:**

Intestinal ultrasound (IUS) is an emerging non-invasive modality for real-time monitoring in ulcerative colitis (UC). Its role in predicting treatment response and long-term outcomes in severe UC remains underexplored.

**Aims:**

To evaluate the accuracy of IUS - particularly bowel wall thickness (BWT), in predicting treatment response and future outcomes in patients with severe UC.

**Methods:**

A comprehensive electronic search was conducted on Medline, Embase and Cochrane through December 2024 to identify prospective cohort studies and randomized controlled trials (RCTs). Primary outcome was the predictive accuracy of ≥ 20% reduction in bowel wall thickness. Secondary outcomes included colectomy rates and steroid response using BWT thresholds (<1–2 mm reduction). Diagnostic test accuracy (DTA) data were synthesized using a bivariate random-effects model (Meta-DiSc 2.0). Odds ratios (ORs) for predictors of nonresponse were pooled using a random-effects model (RevMan 5.4.1)

**Results:**

Four studies were included from 5403 citations reporting outcomes in terms of steroid response and/or need for colectomy. Meta-analysis of two studies assessing a ≥ 20% reduction in BWT showed a sensitivity of 0.83 (95% CI 0.67–0.92) and specificity of 0.77 (95% CI 0.64–0.87) for predicting steroid response. Positive and negative likelihood ratios were 3.66 (95% CI: 1.76–5.56) and 0.22 (95% CI: 0.06–0.39), respectively, indicating strong rule-in and rule-out utility. One study reported that BWT >4.0 mm was associated with an OR of 9.5 (95% CI: 1.5–186) for colectomy at 3 months. A BWT reduction <2 mm was associated with an OR of 3.8 (95% CI: 0.6–75) for colectomy

**Conclusions:**

IUS, particularly BWT assessment, demonstrates promising diagnostic accuracy for predicting steroid response in severe UC and may aid in early identification of patients at risk of treatment failure. Although early data suggest potential in predicting colectomy risk, current evidence is limited by heterogeneity and small sample sizes. Larger, prospective studies are needed to confirm the utility of IUS in long-term risk stratification and treatment optimization

**Funding Agencies:**

None